# Detecting ulterior motives from verbal cues in group deliberations

**DOI:** 10.3389/fpsyg.2023.1166225

**Published:** 2023-05-24

**Authors:** Norah E. Dunbar, Judee K. Burgoon, Xunyu Chen, Xinran Wang, Saiying Ge, Qing Huang, Jay Nunamaker

**Affiliations:** ^1^Department of Communication, University of California, Santa Barbara, Santa Barbara, CA, United States; ^2^Center for the Management of Information, The University of Arizona, Tucson, AZ, United States

**Keywords:** deception detection, verbal deception, interviewing, deceptive messages, structured programming for linguistic cue extraction (SPLICE), Linguistic Inquiry and Word Count (LIWC) features

## Abstract

**Introduction:**

Forensic interviewing entails practitioners interviewing suspects to secure valid information and elicit confessions. Such interviews are often conducted in police stations but may also occur in field settings such as border crossings, security checkpoints, bus terminals, and sports venues. Because these real-world interviews often lack experimental control and ground truth, this investigation explored whether results of non-forensic interviews generalize to forensic ones.

**Methods:**

Organizational espionage was simulated to determine (1) what verbal signals distinguish truth from deception, (2) whether deception in groups aligns with deception in dyads, and (3) whether non-forensic venues can be generalized to forensic ones. Engaging in a mock hiring deliberation, participants (4–5 strangers) reviewed and discussed resumes of potential candidates. Surreptitiously, two group members assigned the role of “organizational spies” attempted to persuade the group to hire an inferior candidate. Each group member presented notes from an interview of “their” candidate, followed by a discussion of all candidates. Spies were to use any means possible, including deception, to persuade others to select their candidate. A financial incentive was offered for having one’s candidate chosen. The interview reports and discussions were transcribed and analyzed with SPLICE, an automated text analysis program.

**Results:**

Deceivers were perceived as less trustworthy than truth-tellers, especially when the naïve players won but overall, deceivers were difficult for non-spies to detect even though they were seen as less trustworthy than the naïve participants. Deceivers’ language was more complex and exhibited an “echoing” strategy of repeating others’ opinions. This collusion evolved naturally, without pre-planning. No other verbal differences were evident, which suggests that the difference between spies and non-spies was subtle and difficult for truth-tellers to spot.

**Discussion:**

Whether deception can be successfully detected hinges on a variety of factors including the deceiver’s skill to disguise and the detector’s ability to sense and process information. Furthermore, the group dynamics and communication context subtly moderate how deception manifests and influence the accuracy of detecting ulterior motives. Our future investigations could encompass non-verbal communication channels and verbal patterns rooted in content, thereby providing a more comprehensive understanding of deception detection.

## 1. Introduction

Deception is a ubiquitous human activity which is used to satisfy goals in human communication. Oftentimes, two interlocutors have goals that are in conflict with one another—one person is trying to create a false belief in another and the other person is trying to accurately judge the credibility of those statements (Burgoon and Buller, 2015). In certain circumstances, these goals are high stakes such as when a suspect is being interviewed by the police in a forensic interview. Forensic interviewing to detect deception typically entails practitioners interviewing suspects to gather information and to determine the veracity of the claims being made through the application of scientific methods and techniques (Shepherd, 2007; Inbau et al., 2013). Police often conduct these interviews, but they can also happen in the field such as at border crossings, security checkpoints, bus terminals, shopping malls, sports venues, and other locations (Vrij, 2014). These real-world interviews often lack ground truth and experimental control, making laboratory and field experiments beneficial if their findings generalize to real-world contexts.

The current investigation was undertaken to explore (1) what verbal signals distinguish truth from deception, (2) whether deception in groups aligns with deception in dyads, and (3) whether non-forensic venues can be generalized to forensic ones. We developed an experimental protocol to assess this possibility. Organizational espionage was simulated to determine whether deception during group deliberations of job applicants could be detected through the verbal content present. Groups of 4–5 participants (strangers) conducted a mock hiring deliberation in which they reviewed resumes of potential candidates and were charged with selecting the best candidates. Surreptitiously, two group members were assigned the role of “spies,” who were ostensibly engaged in industrial espionage. Their goal was to persuade the group to hire a candidate who was objectively weaker than the other candidates. This methodology mirrored that of Dunbar et al. (2014). Group members reviewed the resumes of all candidates. Then each individual presented interview notes from an interview of “their” candidate and presented reasons for their choice of candidate. Spies were instructed to argue for the weak candidates using any means possible, including deception about their qualifications. Non-spies were instructed to hire the most qualified candidate. Both spies and non-spies were given a financial incentive to complete their task. Due to COVID restrictions, discussions took place online using video conferencing. Verbal statements made during the discussion/interview phase of the experiment were captured in verbatim transcripts of the conversations. This article presents the results of this experiment Application of these non-forensic field results to forensic ones were considered.

## 2. Background

### 2.1. Verbal deception in non-forensic settings

Deception detection has been widely studied in various non-forensic investigative interviews, such as security screening, financial auditing, and recruitment interviews. These contexts offer evidence of verbal forms of deception, where organizations and individuals conduct investigative interviews that may not necessarily engage in accusatory interrogation but entail fact-finding investigations (see Vrij, 2008, for an extensive list of references). Examples of these professionals include regulatory investigators, auditors, accountants, human resource professionals, and those who process any kind of application or claim (Shepherd, 2007). Each of these contexts not only speaks to the value of examining verbal clues to deception but also has found relationships between verbal communication and deception detection that are potentially generalizable to other, forensic contexts. One such context that shares the characteristics of investigative interviewing is audit interviews. The narratives gathered from auditor interviews of management during fieldwork are critical forms of audit evidence (Public Company Accounting Oversight Board [PCAOB], 2023). An audit interview study finds that both inexperienced and experienced auditors fail to detect deception at greater than chance accuracy levels (Lee and Welker, 2008). After analysis of publicly available data on question and answer (Q&A) portions of earnings calls, researchers found evidence to support that auditors experientially become more attuned to avoiding false positives than false negatives when detecting deception associated with fraud (Hobson et al., 2017). One of the few investigations of linguistic differences by Burgoon et al. (2016) found differences between manager and analyst language in the Q&A portions of earnings calls. Analysts were more likely to ask questions when interacting with fraudulent firms, and fraudulent managers used less negativity, more dominance, and more hedging language than their non-fraudulent peers (Burgoon et al., 2016; Spitzley, 2018).

A second context relevant to investigative interviewing is security screening, in which security guards must distinguish between innocent travelers and those who may be engaged in unlawful activities. The tremendous flow, brief interactions, and limited human attention make the task a complicated one (Twitchell et al., 2004). Often, interviews must be very brief to reduce the inconvenience to truthful and low risk individuals while producing an efficient flow of travelers through checkpoints. The brevity of such interviews and the sparsity of research on specific linguistic features reduces its applicability to forensic contexts. Nevertheless, both laboratory and field evidence show promise of using automated deception detection systems to identify deceivers at border crossings and security checkpoints using verbal and non-verbal indicators (Nunamaker et al., 2013; Twyman et al., 2015; although see Sánchez-Monedero and Dencik, 2022, for a counter perspective). These automated detection systems can be used in other contexts such as employment interviews and forensic interviewing as well.

Job interviews are a third context in which verbal content may reveal deception and are most akin to forensic interviews in their length, open-ended format and assumption of cooperative communication by truthful respondents (Taylor et al., 2013). Detecting deception from job interviews is difficult but important for human resource management (Roulin et al., 2014), because the poor decision on human capital placement can result in lost productivity and high cost in hiring, recruiting, and training replacements (CareerBuilder, 2017). Identification of reliable human indicators of deception can be leveraged to reduce the risk of bad hires (Twyman et al., 2018, 2020). In the hope of appearing more attractive to employers, more than 90% of job applicants report using some degree of deceit and outright deceptive ingratiation in their interviews (Melchers et al., 2020; see also Roulin et al., 2014; Roulin and Bourdage, 2017; Roulin and Krings, 2020). Job seekers engage in such forms of deceptive misrepresentation as exaggeration and inflation of reported background, and fabrication of skills and experiences (Weiss and Feldman, 2006; Levashina and Campion, 2007). While the bulk of research on deception in job interviews has targeted non-verbal cues, recent studies have shown that verbal cues are more diagnostic and easier for practitioners to reliably use than non-verbal cues (Vrij, 2019). A recent experiment with automated job application systems indicated that word complexity was lower, and the rate of adverbs was higher, for deceptive than truthful responses (Twyman et al., 2020).

These foregoing bodies of research may be applicable to practitioners in a variety of non-forensic contexts as well as forensic ones. To the extent that deception functions in the same fashion in both, the bodies of research collected in several meta-analyses (DePaulo et al., 2003; Aamodt and Custer, 2006; Hartwig and Bond, 2014; Hauch et al., 2015) and summaries of verbal and non-verbal signals of deceit (Sporer and Schwandt, 2006; Burgoon et al., 2021) may generalize more broadly to include forensic contexts.

### 2.2. Deception by individuals versus groups

In the typical deception experiment, like those that use a typical cheating paradigm or a mock crime scenario, an actor will be randomly assigned to tell the truth or lie so that the researchers can establish what is called “ground truth” and know precisely who the liars are (Levine, 2020). Interviews to detect deception in research settings most often occur one-on-one but in the real-world context, groups often work on tasks together. As such, groups of people are responsible for flagging and reporting suspicious behavior. Research has shown that, on one hand, groups, especially established groups with prior interaction, can detect deception more accurately than individuals (Klein and Epley, 2015; McHaney et al., 2018; Hamlin et al., 2021). On the other hand, group size does not significantly affect detection accuracy (Hamlin et al., 2021). Multiple individuals may also deceive collectively [e.g., interviewing multiple suspects simultaneously in Vernham and Vrij (2015) and Vernham et al. (2016)]. However, research on deception in groups is still somewhat limited (for exceptions, see Hung and Chittaranjan, 2010; Wright et al., 2012; Zhou et al., 2013; Vernham and Vrij, 2015; Vernham et al., 2016), and it is common to fail to differentiate between research from dyads and research from groups. However, such generalization is often wrong, for several reasons. First, as groups grow in size from 2 to 20, individual degrees of engagement and participation may decline. With that decline comes a weakening of involvement with the group’s topics of discussion, and a heightened presence of social loafing (Latané et al., 1979; Alnuaimi et al., 2010). It is easy for a group member to lose interest if the topics do not relate to that individual. As interest wanes, so does attention (Deci and Ryan, 1985). Unlike dyads, in which individuals must maintain at least a semblance of interest in what the interlocutor is saying, group settings allow group members’ attention to wander so that measures of their interest become increasingly unequal (Kerr and Bruun, 1983).

Unlike in dyads, group members may also develop coalitions and clique groups, forming collusion with one another, especially when their self-interests diverge from the group at large (Komorita and Kravitz, 1983). Moreland (2010) argues that individuals are likely to experience stronger and more negative emotions in dyads than in groups. Deception is a case in point where members hold ulterior motives and engage in counter-behaviors (Buller and Burgoon, 1994). Covert and sly actions become much more likely as the group size grows. Other qualitative relationships also differ in dyads and groups of different sizes. For example, the complexion of affiliative feelings changes, group cohesion suffers, and information exchange becomes uneven as the group grows larger (DeSanctis and Gallupe, 1987; Wheelan, 2009).

Physicality changes as well when moving from dyads to groups. Whereas face-to-face dyads are typically within close proximity to one another–usually 2–4 feet, in groups, their distance from one another varies. It might seem likely that those who are adjacent to one another talk more often, and such proximity does foster some conversation, but research on small group interaction has shown that those who are directly opposite one another have the most interaction (the so-called “Steinzor effect,” Steinzor, 1950). Seating arrangement can also dictate conversational distance, placing group members at different distances from the leader. In leaderless groups, seating arrangements can influence who becomes the leader: those at the head of the table or opposite the most others are more likely to be leaders (Burgoon et al., 2021). People working in teams or groups also sometimes “talk to the room” and direct comments to the group as a whole rather than one person in particular (Dunbar et al., 2021).

Information processing in groups also becomes more taxing. Attending to what multiple group members say, plus watching and listening for non-verbal signals from multiple members and allowing multiple members to have turns-at-talk, becomes more cognitively demanding becomes more cognitively demanding as the amount of information dramatically increases (Sweller, 2011; Van Der Zee et al., 2021). The result being that groupwork is less pleasurable and more tiring than dyadic deliberations. It also means that increasing cognitive complexity can make it more difficult for group members to detect deception among one another.

Finally, groups afford members the opportunity to “lay low” and speak very little. They can choose to ride on others’ coattails and adopt a quiet communication style, something that is impossible in dyads. Interviewees must take as many turns-at-talk as the interviewers. By hanging back, deceivers may devote more energy to surveilling others.

The combination of all the foregoing factors produces a complexity that is absent from dyadic interactions, making predictions of group outcomes more uncertain the larger the group size. Put differently, groupwork is a different animal than dyadic work. This does not mean we cannot learn from the vast research on deception detection in dyads and apply that knowledge to groups, however (Williams, 2010). Forensic interviews among multiple individuals implicated in the same crime become a complicated tapestry in which the various strands of the storyline must be untangled. Each person’s strand may introduce a different color and warp. The investigator’s task becomes determining which ones go together and corroborate each other rather than producing a collusive, accurate rendition.

### 2.3. Research questions

RQ 1: Are naïve members of a group able to detect deception from those with malicious intent?

RQ 2: Can linguistic cues of quantity, diversity, complexity, dominance, certainty and personalism differentiate insiders’ and non-insiders’ language use to provide verbal cues to deceit?

RQ 3: Is deception evident from patterns of interaction among group members?

## 3. Materials and methods

### 3.1. Participants

We conducted experimental sessions with participants recruited from two large public universities in the Western US to engage in group interactions that simulated hiring decisions. The experiment was multi-phased, including review of resumes and interview notes, individual monologs, and group discussion. When there were not enough participants to form a group, we instructed those who showed up (*N* = 26) to perform an alternative task described in section “4.1. Alternative task: ranking the candidates.” Participants (*N* = 109; 72 females, 35 males, and two who did not report gender) formed 22 group experiment sessions. One session had four participants, while all other sessions had five participants each. Among these participants, 55.0% were white; 19.3% were Asian; 9.2% were Hispanic/Latinx; and 3.7% were Black. Multiracial and other participants accounted for 9.2 and 3.7%, respectively. Average age was 21.3 years old (SD = 2.1; min = 18; max = 31). A total of 79.8% were native English speakers. Participants received $10 USD or extra course credit to compensate them for their time.

### 3.2. Design

The methodology mirrored that of Dunbar et al. (2014), which used chat conversations. The experiments were held on an online platform for synchronous video communication. In each session, a group of four to five participants simulated a hiring committee and worked together to identify the best candidate to hire, based on the candidates’ qualifications. A trained research assistant facilitated each session by presenting videotaped instructions and following a standard script to ensure the consistency of experimental protocols across sessions.

After signing into the online platform, participants completed the consent form and demographic information. Following a randomized order, they introduced themselves to other participants. Each then rated the other participants’ trustworthiness on four items: whether they thought the individual was dishonest, reliable, deceitful, and trustworthy. Ratings were on a five-point Likert scale and reflected participants’ baseline perception of one another.

Next, they were all given a job description and five resumes from hypothetical candidates. The resumes included the candidates’ education, employment history, and other information (e.g., skills, awards, and interests). Two of the resumes were designed to show preferable characteristics and have high quality. In contrast, two other resumes were unprofessional and less relevant to the job description and thus had low quality. One resume was of medium quality. Dunbar et al. (2014) pilot-tested the resumes with experts who unanimously agreed on the strongest and weakest resumes. For the four-person group, one of the high-quality resumes were not distributed. Without being told which resumes were of high, medium, and low quality, participants were instructed to read through the resumes and rank the candidates based on how well suited they were for the job. A rank of one indicated the candidate was thought to be the best candidate, while a rank of five meant they were the worst candidate. The job description and resumes were available to the whole group.

**FIGURE 1:**
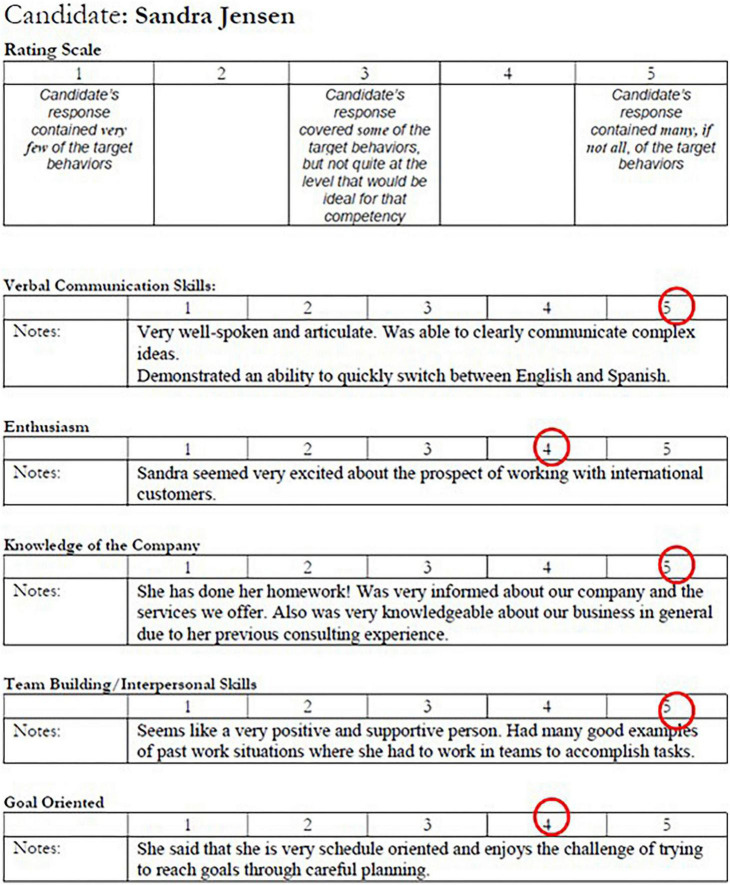
The sample interview note of one of the top hypothetical candidates, Sandra Jensen. Sandra scores four to five in all the five metrics.

**FIGURE 2:**
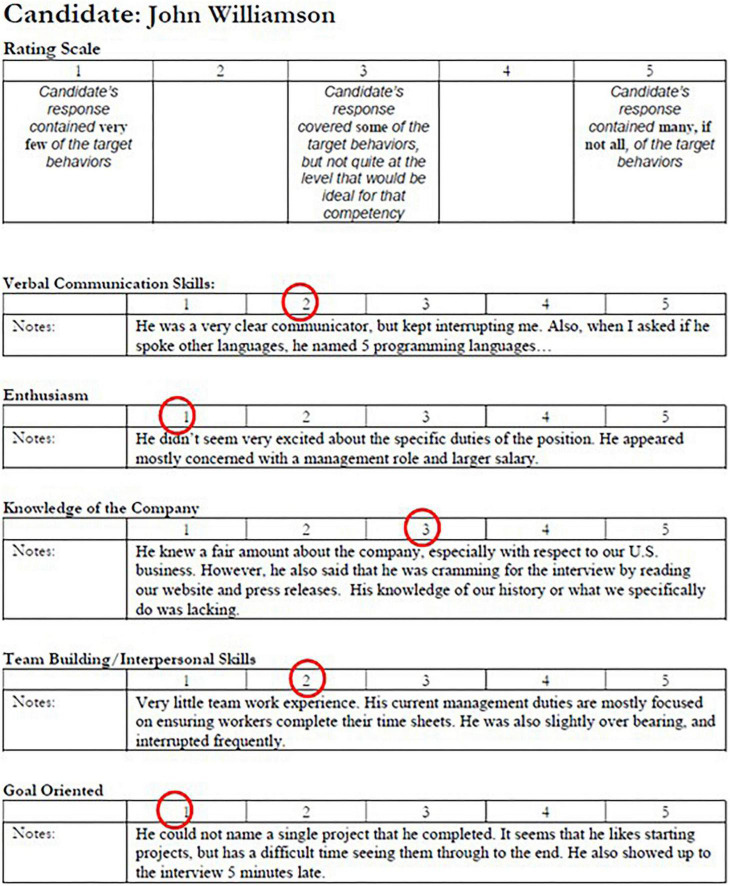
The sample interview note of one of the worst hypothetical candidates, John Williamson. John scores one to three in all the five metrics.

Two participants were randomly assigned to be deceivers, and the rest of the participants were assumed to be truthful. Each participant was instructed to review one interview note which documented one candidate’s interview performance and to prepare a summary for the other committee members. The truth-tellers each received an interview note of one of the candidates with high- or medium-quality resumes, while the deceivers’ interview notes corresponded to the low-quality resumes. The interview notes listed the evaluation of candidates’ verbal communication skills, teamwork and interpersonal skills, enthusiasm, knowledge of the company, and goal-orientation. Two sample interview notes are presented in Figures 1, 2. The candidates with the high-quality (or low-quality) resumes also performed well (or poorly) in the interviews and were therefore the best (or worst) candidates. The candidate with the medium-quality resume had mediocre interview performance. Because each participant only reviewed one candidate’s interview note, an interview note was only known to one participant.

The deceivers were informed that they were corporate spies from a competitor company and their goal was to persuade the group to hire another spy who did not qualify for the position. If either one of the two worst candidates was hired, the deceivers won. In contrast, the truth-tellers were instructed that, in order to win, the group should hire the best candidate. By definition, the best candidate was either one of the top two candidates. For the four-person group, there was only one best candidate, because the other top candidate’s resume and interview note were not distributed. Truth-tellers did not know that some participants would advocate for unqualified candidates. Both deceivers and truth-tellers were told they would each vote for the candidate to hire at the end of the experiment, and winners would receive a five-dollar bonus.

**FIGURE 3:**
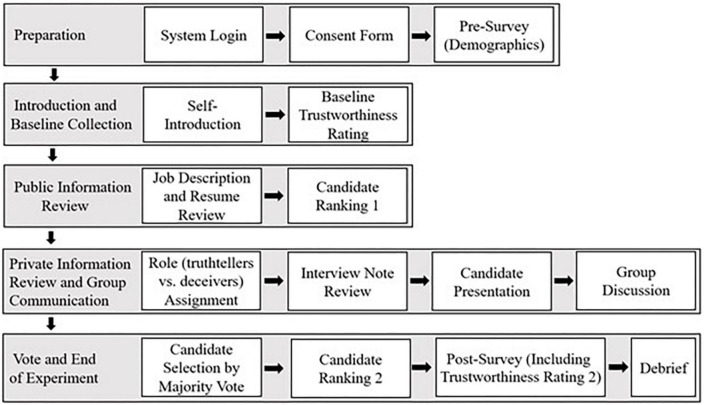
Summary of the experiment procedures.

Participants were given up to 1 min to summarize their interview notes. They could also include details from their candidate’s resume if they chose to. Presentations followed a randomized order. Then the group spent 5 min discussing the best candidate to hire. Deceivers were told they could embellish the interview note during the candidate presentation and group discussion. Therefore, although the truth-tellers knew which candidates had the strongest and weakest resumes, they could be given false information about the candidates’ interview performance and persuaded to select an unqualified candidate. After the discussion, participants voted for the best candidate, and the candidate with the majority vote would be hired. Participants ranked the candidates again before the facilitator announced the voting result. Finally, participants filled out a post-experiment survey and rated the information they gave to the group on its completeness, detail, accuracy, etc., on a five-point Likert scale. Participants also rated each other’s trustworthiness on the same four items (i.e., dishonest, reliable, deceitful, and trustworthy) again. Figure 3 summarizes the experiment procedures.

### 3.3. Self-reported perceived trustworthiness

After the self-introduction, participants rated baseline perceptions of one another on four items: whether they thought the individual was dishonest, reliable, deceitful, and trustworthy. The dishonest and deceitful items were reverse coded. A higher number reflected honesty and truthfulness. Two attention check questions were embedded. Three participants failed both questions and thus did not pass the attention check. Their ratings were removed. Cronbach’s alpha of the four items was 0.794. The average of the four items was the trustworthiness score given by a rater to a ratee. We measured every participant’s baseline perceived trustworthiness by averaging the trustworthiness scores they received.

In the post-experiment surveys, participants were asked to rate each other on the same items. Ratings from one participant who did not pass the attention check were removed. Deceivers’ ratings were also removed because they knew who was deceptive. Cronbach’s alpha of the four items was 0.859. For every participant, we measured the perceived trustworthiness by averaging their trustworthiness scores given by the truth-tellers.

### 3.4. Linguistic tools and measures

To detect verbal cues to deceit, we manually transcribed participants’ speech and employed SPLICE (Moffitt et al., 2012), an automated linguistic analysis tool, to extract language features. The language features of interest were quantity, diversity, complexity, dominance, certainty, and personalism. The definitions of these features are listed in Table 1. These composite measures combine several linguistic features and are meant to offer a more advanced tool to complement the frequently used Linguistic Inquiry and Word Count (LIWC; Pennebaker et al., 2007). The tool incorporates features of language used in previous analyses of language such as the General Architecture for Text Extraction for parsing and the Whissell dictionary for affect-related terms (e.g., Bradac and Mulac, 1984; Whissell et al., 1986; Bontcheva et al., 2002; Cunningham, 2002). The ones chosen are ones that have emerged in prior analyses of linguistic features and meta-analyses (e g., Qin et al., 2004; Zhou et al., 2004; Burgoon and Qin, 2006; Hartwig and Bond, 2014; Hauch et al., 2015; Burgoon et al., 2016). Quantity refers to the number of words, which commonly emerges in tests of language features and has been found to be negatively associated with deception (Hauch et al., 2015). Diversity is the percentage of unique words. Complexity combines lexical, syntactic and semantic measures. Deceivers are predicted to use more redundant, simpler, less diverse, and complex language unless obfuscating (Vrij et al., 2011; Hauch et al., 2015). Dominance includes a variety of indicators signaling one-up status. Deceivers’ dominance is context-dependent (Dunbar et al., 2014, 2021). When attempting persuasiveness, deceivers become dominant; when attempting to evade detection, deceivers choose a non-dominant demeanor. Certainty is measured by the ratio of hedging words, uncertainty quantifiers, and uncertainty terms. Deceivers express more uncertainty (Zhou et al., 2004) unless they have planned or rehearsed their deception in advance (Burgoon et al., 2016). Personalism encompasses first-person pronouns versus third- and second-person pronouns. Deceivers are predicted to avoid first-person pronouns (Pennebaker et al., 2003, 2007; Hauch et al., 2015).

**TABLE 1:** Linguistic composites and definitions.

Linguistic variable	Description	SPLICE variable
Quantity	The number of words in a passage of text	Number of words
Diversity	The percentage of unique words in a passage of text	Lexical diversity
Complexity	The syntactic and linguistic complexity of a passage of text	Complexity composite
Dominance	The percentage of dominant turns-at-talk in a passage of text	Dominance ratio
Uncertainty	The ratio of hedging words, uncertainty quantifiers and uncertainty terms in a passage of text	Hedging and uncertainty ratio
Personalism	The use of personal pronouns. First-person plural pronouns (e.g., we) are the most personal.	Ratio of first-person plural pronouns to total number of words

### 3.5. Conversational pattern analysis

To further discover the differences between successful and unsuccessful deception, we analyzed the group conversational patterns by manually abstracting content within each verbal turn into a set of entity transition sequences. As a preliminary analysis, one of our researchers conducted one round of manual coding on the transcripts of each group’s discussion section. This involved categorizing the speech acts present in the data and identifying their directionality, including the speaker(s) and addressee(s) involved in each speech act, as well as the candidate(s) discussed. Another researcher examined the codes with the previous coder’s coding schema (see Appendix) and utilized entity grids to visualize them. Further analysis was conducted to identify conversational patterns within the data. Specifically, our investigation focused on the manner in which spies participated in the group discussion, including their level of engagement (e.g., actively diverting the conversation or passively following its flow) and the extent of their collaboration with one another (e.g., supporting each other’s candidate or challenging each other’s arguments to bolster their credibility). After transforming the group discussion content into a set of entity transition sequences, we adapted the Entity-Grid Discourse Representation (entity grid) matrix (Barzilay and Lapata, 2008) to capture the micro conversational episodes. An entity is originally defined as a class of co-referent noun phrases that refer to or symbolize the same thoughts or reference (Barzilay and Lapata, 2008). In our study, key entities include the targeted subject (i.e., the job candidate who is being discussed) and the targeted group member (i.e., to whom the current speaker is responding). Given the dynamic nature of conversation, we added annotations that signal the speakers’ attitude with the speech act codes (e.g., supporting or disparaging a candidate and whether or not they agreed with a specific group member). In our matrix, each column represents a single entity and each row represents the content of a verbal turn. An empty cell indicates that a verbal turn represented by the row does not include the entity represented by the column. We also color-coded each column to identify the speaker.

## 4. Results

### 4.1. Alternative task: ranking the candidates

Twenty-six participants who did not form a group conducted an alternative task. These participants were asked to review the job description, candidates’ resumes, and interview notes and rank the candidates based on all the information. A rank of one indicated the best candidate, and a rank of five denoted the worst candidate. This ranking complemented the main experiment for validating the top and worst candidates. Recall that participants in the main experiment provided the first ranking of candidates prior to the role (truth-tellers versus deceivers) assignment based on only the job description and resumes. The candidates’ average rankings given in the alternative task are shown in the first row of Table 2. These participants were able to correctly identify the top, medium, and worst candidates, showing that the resumes and interview notes were properly designed.

**TABLE 2:** Candidates’ average ranking ranked by participants in the alternative task, truth-tellers, and deceivers.

Time of ranking	Participant role	Top candidate 1	Top candidate 2	Medium candidate	Worst candidate 1	Worst candidate 2
At the alternative task	Alternative task	1.35 (0.85)	2.19 (0.69)	2.77 (0.71)	4.27 (0.83)	4.42 (0.81)
After resume review and before role assignment	Truth-tellers	2.00 (1.03)	1.69 (0.92)	3.23 (0.98)	3.48 (1.00)	4.60 (0.81)
	Deceivers	1.84 (0.81)	1.86 (1.03)	2.98 (1.00)	3.75 (1.01)	4.57 (0.85)
	All	1.94 (0.95)	1.76 (0.96)	3.13 (0.99)	3.59 (1.01)	4.59 (0.82)
After role assignment and group discussion	Truth-tellers	2.05 (0.91)	1.94 (0.81)	**4.22** **(0.70)**	*2.69* *(1.32)*	4.11 (1.24)

The average rankings are outside the parentheses, and the standard deviations are within the parentheses. A rank of one indicates the best candidate, and a rank of five indicates the worst candidate. After role assignment and group discussion, the medium candidate’s ranking (in bold) dropped to the last, while one of the worst candidates moved up and ranked the middle (in italics).

### 4.2. Manipulation checks

Candidate rankings also occurred in the main experiment in two stages based on different information. Participants were first asked to rank the candidates’ resumes before the role assignment. They were asked to rank the candidates again after the group discussion, at which time they had received information on resumes and interview notes. We report these two rankings in Table 2. Deceivers’ rankings after the group discussion are omitted because they were aware of their own deception. As expected, truth-tellers’ and deceivers’ rankings were similar before role assignment, and both truth-tellers and deceivers were able to identify the top, medium, and worst candidates. However, after the group discussion, the medium candidate (in bold) dropped to the last, and one of the worst candidates (in italics) was ranked the middle. Therefore, the truth-tellers perceived the qualifications of one of the worst candidates to be better than they actually were, and our manipulation was successful.

Another manipulation check in the post-experiment surveys asked participants to rate the information they gave to the group about their candidate. We conducted *t*-tests to compare truth-tellers’ and deceivers’ ratings and report the results in Table 3. Truth-tellers rated their information as more complete, detailed, believable, accurate, clear, precise, true, truthful, exact, and helpful to the group, while deceivers rated their information as more incorrect, uninformative, and overstated. These results indicate that the manipulation was successful.

**TABLE 3:** Participants’ self-ratings of the information they gave to the group.

Participant role	Complete***	Detailed**	Believable***	Accurate***	Clear***
Truth-tellers	4.17 (0.76)	3.88 (0.91)	4.46 (0.71)	4.52 (0.64)	4.25 (0.69)
Deceivers	2.86 (1.34)	3.16 (1.24)	3.66 (1.22)	2.09 (1.27)	3.11 (1.32)
**Participant role**	Precise***	**Persuasive**	**Convincing**	True***	Truthful***
Truth-tellers	4.03 (0.81)	4.02 (0.93)	3.88 (0.99)	4.75 (0.56)	4.75 (0.50)
Deceivers	2.16 (1.26)	3.64 (1.31)	3.61 (1.20)	2.00 (1.18)	1.89 (1.15)
**Participant role**	Exact***	Incorrect***	Uninformative**	Overstated***	Helpful to the group***
Truth-tellers	4.40 (0.75)	1.72 (1.05)	1.86 (1.18)	2.92 (0.92)	4.82 (0.56)
Deceivers	1.84 (1.14)	3.93 (1.15)	2.52 (1.21)	3.93 (1.00)	1.61 (1.06)

The average ratings are reported outside the parentheses, and the standard deviations are reported within the parentheses. t-Tests are conducted to compare truth-tellers’ and deceivers’ ratings. **p < 0.01, ***p < 0.001.

### 4.3. Analytical responses to research questions

#### 4.3.1. RQ 1

To study whether the naïve participants were able to detect the deceivers, the deceivers’ perceived trustworthiness was compared against that of the naïve participants. If the deceivers were perceived as less trustworthy than the naïve participants, we concluded the naïve participants implicitly were able to detect deception. A non-parametric Mann–Whitney means test in the post-introduction survey (prior to the deception manipulation) indicated no significant difference in perceived trustworthiness between the deceivers and the naïve participants (U-statistics = 1,455.0, *p* = 0.880). The same test yielded a significant difference in perceived trustworthiness between the two parties in the post-experiment survey (U-statistics = 1,009.5, *p* = 0.009). The naïve participants’ aggregated trustworthiness score (mean = 3.92, SD = 0.51) was higher than that of the deceivers (mean = 3.67, SD = 0.52). Therefore, the naïve players were able to discern deception and indirectly detect the deceivers.

As the awareness of deception affects decision making, a comparison of trustworthiness that accounts for the deception outcome may further reveal in which circumstances the naïve participants performed better at detecting deception. In half of the groups, the deceivers won. We replicated the comparison of trustworthiness when the espionage was successful and when it was not. When the deception was successful (the deceivers won), no significant difference in perceived trustworthiness was found between the deceivers and the naïve participants (U-statistics = 312.5, *p* = 0.49). However, when the naïve participants won, the deceivers were perceived as significantly less trustworthy than the naïve participants (U-statistics = 190.0, *p* = 0.003). The results show that in only half of the groups, suspicion was triggered and affected decision making.

#### 4.3.2. RQ 2

Multiple mixed-effects linear regression models were developed to address RQ 2. The experiment role (deceivers versus naïve participants) was the main predictor variable. The regression also considered the interaction between the experiment role and the experiment outcome (deceivers won versus naïve participants won). A participant’s gender, native language, and previous experience in hiring activities were controlled. Considering the nested design of the data collection, a mixed-effects specification was adopted using the group identifiers as the random-effect term. Table 4 presents the regression results.

**TABLE 4:** Multiple regression analysis of deception regressed on linguistic variables (regression coefficients and *p*-values reported).

Dependent variable	Number of words	Lexical diversity	Complexity composite	Dominance ratio	Hedging and uncertainty ratio	First-person plural ratio
Deception	0.420 (0.988)	−0.000 (0.999)	0.1277** **(0.029)**	−0.004 (0.875)	−0.009 (0.558)	0.004 (0.181)
Winner	−13.923 (0.588)	0.012 (0.751)	0.000 (0.995)	0.018 (0.425)	−0.008 (0.559)	0.000 (0.893)
Male	25.410 (0.232)	−0.006 (0.828)	−0.046 (0.281)	0.004 (0.866)	−0.009 (0.431)	0.002 (0.470)
Native English speaker	11.068 (0.663)	0.002 (0.947)	−0.056 (0.284)	0.035 (0.110)	0.014 (0.300)	−0.004 (0.125)
Hiring experience	54.162** **(0.037)**	−0.064** **(0.056)**	−0.045 (0.396)	−0.027 (0.210)	−0.002 (0.879)	−0.002 (0.484)
Deception* winner	17.576 (0.662)	−0.039 (0.443)	−0.105 (0.191)	0.002 (0.964)	0.020 (0.332)	−0.006 (0.166)
Fitness (AIC)	1,258.261	−82.822	11.278	−169.744	−265.429	−576.314

The bold values indicate that the coefficients are statistically significant. **p < 0.05.

The regression models show that the deception manipulation only produced differences in language complexity, with the deceivers’ speech being more complex compared to the naïve participants. Secondly, hiring experience increased a participant’s language productivity and reduced lexical diversity. Overall, no other verbal features except language complexity exhibited differences between the deceivers and the naïve participants. The control variables such as gender and native language also did not explain much variance in the linguistic variables. We found an increase of complexity in the deceivers’ language, which may be accounted for by the preparation of the deception.

Though not many linguistic variables reliably manifested hiring espionage, multiple linguistic measures when combined may predict deception (see Hartwig and Bond, 2014, regarding combinations of non-verbal cues). To test this assumption, a discriminant analysis was performed. All linguistic features included in the regression analysis were used to differentiate the truth-tellers and the spies. To better explore the feature space and identify the most effective discriminant function, all subsets of the six linguistic variables were also tested in a random-split training and testing process. Specifically, 17 groups were randomly selected to train a discriminant function. The discriminant function was then evaluated on the remaining five groups. The random split was repeated 1,000 times for each set of predictors. The average classification accuracy was reported to evaluate the discriminant power of the corresponding linguistic features. The prediction accuracy of the discriminant function that contained all six linguistic features was consistently below 60%, which is the percentage of truth-tellers in our experiment. Among various subsets, the highest accuracy, 0.636, was achieved by the discriminant function that used Lexical Diversity and Complexity Composite as its predictors. However, this accuracy level was still far from being satisfactory for detecting infiltrators. Therefore, from both the regression and the discriminant analysis, we suggest that language style provides very weak utility for detecting hiring espionage.

#### 4.3.3. RQ 3

Our preliminary analysis of the conversation patterns indicates some systematic differences between spies’ and non-spies’ sequences. To reach an agreement on the best candidate to hire, group members needed to express their opinions about the job candidates, question one another about their opinions and even debate their views. Therefore, it is important to look into the transitional sequences where a group member expresses an opposite opinion of the current candidate or switches the focus to another candidate. From the entity grid matrix, we highlighted these sequences as well as categorized the speakers who initiated the transitions (spies versus non-spies) and the targeted candidates (low-quality ones versus others).

Among all experiment groups, non-spies more frequently initiated these transitions that changed the discussion direction. In comparison, spies were more passive and vigilant. From the entity grid matrix, we found 172 occurrences of these transitional sequences from 18 groups; 63 were initiated by a spy and non-spies initiated the rest. This could potentially be explained by the difference between truth-telling and deception. Non-spies can express their opinions more freely by pointing out both the strengths and weaknesses of a candidate, while spies needed to be more cautious with what they said about a candidate because they wanted to selectively present certain features (e.g., strengths) of the worst candidates.

However, we also noticed that this tendency was more salient in groups where spies successfully persuaded the group to choose one of the worst candidates (only 29 out of 85 transitions were made by spies). In a group setting, it may be easier to persuade other members when a spy is echoing others’ opinions instead of being the first one to propose a different opinion. This can also be an effective strategy when two spies are collaborating (e.g., one first supports the other’s assigned candidate and the other echoes that). Our further examination of the ten successful deception groups’ sequences provided some support for a strong “echoing” effect. In 4 out of the 10 groups, the first transition to show favor for the finally chosen worst candidate was made by a non-spy. In four other groups, although non-spies did not make the first transition, they oriented the discussion back to the worst candidate later after the topic was switched to other candidate(s). The collaboration between the two spies (e.g., supporting the other’s assigned candidate) appeared in six groups. Among the eight groups that failed the deception task, although collaboration between spies still happened in four groups, spies in seven out of eight groups made the first transition, possibly to show favor for their assigned candidate by themselves or to fill the conversational void if the non-spies did not speak up. In summary, by patiently waiting for others (including the other spy) to bring up the assigned worst candidate, spies significantly increased their chance of successful deception. One of the potential reasons is that spies can create a supportive atmosphere covertly in the group for one of the worst candidates in this way.

## 5. Discussion

The current special topic explores the role of language in revealing deception in forensic contexts. The first most obvious question in our investigation is, what facets of language distinguish truth from deception and do they differ in non-forensic as well as forensic contexts? Relatedly, does deception in dyads, which is the prototypical communication format for forensic interviews, differ from when the format is groups? Because different considerations emerge when the number of participants expands from the two-person dyad to the multi-person group, another question that suggests itself is, do additional aspects of interaction reveal anything else about veracity? These are the questions that animated our investigation.

Whether and to what extent the alert was triggered may depend on the additional aspects of deceivers’ ability to deceive and the naïve participants’ ability to detect deception. Poor liars perhaps were easily spotted by skillful lie detectors, and therefore failed the task. Experienced deceivers may have disguised deception as effective persuasion and got away with it. Revealing the determinants of the ability to detect deception, however, needs further investigation.

Participants, serving as a mock hiring committee, simulated a multi-phase screening process that included presenting the qualifications of a single candidate then engaging in a group deliberation about the five candidates under consideration. Two group members were incentivized to support low-quality candidates and would be rewarded if one of their candidates was chosen. Results using automated tools for linguistic analysis showed that deceivers (those misleading the group about the quality of their candidate) were trusted less than truthful participants. Something in their verbal and/or non-verbal demeanor did not engender trust. However, their individual language use was not particularly revealing. Only the complexity of their speech differed, whereas other linguistic properties did not. By definition, complexity was measured by a composite of polysyllabic words, singular or mass nouns, plural nouns, coordinating conjunctions, subordinating conjunctions, prepositions, commas, and average sentence length (Burgoon et al., 2016). A greater value of this variable indicates a higher level of syntactical and linguistic complexity of the sentence. The prevailing view in the deception literature is that deceivers’ language is less complex compared to the truth-tellers’, as producing complex sentences and fabricating false statements would compete for finite processing capacity. In our study, the deceivers, rather than truth-tellers had more complex speech, possibly due to the experiment design and the deceiver’s efforts to obfuscate their position (Markowitz and Hancock, 2016; Markowitz et al., 2021). As the espionage was anticipated, the deceivers could focus on developing arguments in support of specific candidate(s). They were saved from the effort of selecting a candidate at their own discretion. The reduction in cognitive effort and time could allow more mental effort invested in mental searching for more convincing language. The well-thought-out language might have been more sophisticated and complex compared to the naïve participants’ language. Alternatively, the complexity may have introduced obfuscation in support of the low-quality candidates. By using verbalisms to describe the weaker candidates, the descriptions introduced ambiguity. This ploy is often ascribed to politicians’ intent on avoiding clear, concrete answers to questions. Other linguistic variables did not yield significant differences between the deceptive and naïve participants. Possibly, deceivers were able to match the language of naïve group members to achieve their goals (Richardson et al., 2014). Beyond the individual verbal features, our analysis suggested that interaction patterns among group members were more telling. Examination of transition matrices revealed collaboration and an “echoing” effect that enabled moving the deliberations to discuss the poorest candidates. These initial exploratory analyses suggest some subtle ways in which deception was revealed.

Another purpose of the current investigation was to assess the generalizability to a new context of deception cues from our previous group deception experiment. The previous experiment entailed a mock “Resistance” game by groups of villagers warding off spies who intended to do harm. Truth-tellers in that experiment rated spies as less trustworthy over time, whereas ratings of villagers’ trustworthiness slightly increased in later rounds of the experiment (Burgoon, 2021). Both experiments show that truth-tellers can indirectly discern deception in groups. Comparisons of the linguistic content of (truthful) villagers to deceptive spies showed that deceivers were more constrained, echoing the content of the other spy and using more complex language that obfuscated rather than clarified. Comparatively, the deceivers in the Resistance experiment had more distinguishing verbal “tells.” They spoke less than the truth-tellers. The Resistance deceivers could adopt the “flight” strategy and deceive only when necessary. However, the Resume deceivers had to be more proactive in order to promote the less favored candidates. Clearly, the context shapes verbal content and style and argues for conducting experiments in the context of interest rather than “borrowing” conclusions from other investigations (see Markowitz et al., 2023).

In meta-analysis, deceptive accounts have shown to be less plausible, less intimate (or immediate), more uncertain and more repetitive than truthful statements (DePaulo et al., 2003). In our analysis, the paucity of deception findings has an important implication for deception: deception is very difficult to detect but easy to perpetrate, especially in a group where personnel may be colluding. In general, groups afford deceivers more latitude in which to operate. They may mimic or echo others’ behavior patterns. They may choose to be more silent, passive members of the group—the so-called “hiding in the weeds” strategy—while being vigilant about others’ reactions. Or, they might attempt to be persuasive, especially later in the group’s deliberations. Unlike dyads, in which each person has conversational responsibilities, groups are a great place to hide one’s intentions.

In addition to these deliberate actions by individuals, group dynamics can also influence deception outcomes. The success key for the spies also involves whether they can enlist others to back their candidate or others are more persuasive in advocating for different candidates. For example, when there is a convincing opinion leader who advocates for a strong candidate, spies are likely to face more resistance when voicing support for another candidate, which decreases their chance to win.

The novel protocol we developed had the advantage of mimicking the realistic, complex characteristics of insider threat communication but also had the disadvantage of lack of experimental control. Researchers must decide whether to privilege ecological validity or experimental control and the artificiality it brings. This is a common problem facing communication scholars attempting to create realistic circumstances that elicit valid behavior.

One direction for future research is capturing and analyzing non-verbal cues such as a speaker’s head nods, vocal hesitations and response latencies. Head nods often accompany persuasiveness, whereas hesitations and long pauses before responding detract from it. A multimodal approach of looking at non-verbal as well as verbal patterns of discourse may account for more variance. There are many possible combinations of non-verbal and verbal features that may enlighten insider espionage. Another direction is to dig deeper into the linguistic realm through content or conversational analysis. There are many other linguistic variables such as affect and obfuscation that could be tested, but it is reasonable to assume that verbal features beyond the lexical and syntactical level, such as the content or patterns of conversation, may provide another clue to deceit that could be automatically generated.

This experiment is not without weaknesses. Group size was a problem. Too often we did not have enough participants to fill out multiple groups of five and could only have one group at a time. Additionally, this small sample size, with group as the unit of analysis, underpowered our statistical analysis. This weak power may have accounted for some of the non-significant results. Inasmuch as the current corpus is underpowered, collecting more data perhaps will rectify this problem.

Data collection of groups requires tremendous planning and coordination, which partly explains our small sample size and thus the prevalence of null results in this study. The goal was to create groups large enough to deviate from dyads. However, we recommend if this experiment is replicated, to use a more manageable group size of four.

Collecting additional linguistic variables is also advisable. Previous investigations of deceptive language have often recommended combining tools like SPLICE and LIWC, once overlapping variables are removed (e.g., Jensen et al., 2011; Hauch et al., 2015; Burgoon et al., 2016).

## 6. Conclusion

In real-life contexts such as hiring committees, group members often interview one another during discussion to probe their decisions, and deception may occur and interfere with the process. Future research should probe further how deception transpires verbally in groups, because previous findings based on dyadic deception research may not apply to group settings. We conducted an online experiment in which groups of participants simulated a hiring committee with two deceivers covertly promoting unqualified candidates. We analyzed participants’ linguistic features with SPLICE and their conversational patterns with entity grid matrices. The deceivers were less trusted than the naïve participants, especially when the deception was unsuccessful, showing that naïve group members could indirectly discern deception. But more overt verbal measures of deception did not materialize, indicating that in general, deceivers evaded detection. Exceptions were that deceivers used more complex language than naïve participants. Otherwise, we did not find significant differences in language quantity, diversity, dominance, certainty and personalism between spies (deceivers) and naïve (truth-telling) group members. Although disappointing, the results hint at the difficulty of discerning deception from verbal cues. The problem may have been the focus on individual rather than discursive patterns of behavior. Language analyzed at the discourse level revealed an “echoing” strategy by deceivers that facilitated collusion and garnered support, something which could be examined further in future studies.

The null results in this investigation indicate that it is important for deception research to account for group size and context (e.g., groups versus dyads, different domains of tasks) to uncover verbal features that are valid in forensic and other non-cooperative circumstances.

## Data availability statement

The raw data supporting the conclusions of this article will be made available by the authors, without undue reservation.

## Ethics statement

The studies involving human participants were reviewed and approved by the Human Subjects Committee at University of California, Santa Barbara. The patients/participants provided their written informed consent to participate in this study.

## Author contributions

ND and JB contributed to the study design, rationale, and literature review as well as the discussion and analysis. XC, XW, SG, and QH contributed to literature review, analyses, results, and discussion. JN supervised data collection and reviewed/edited the final submission. All authors contributed to the article and approved the submitted version.

## Author disclaimer

The views and conclusions contained in this document are those of the authors and should not be interpreted as representing the official policies, either expressed or implied, of the Army Research Office or the U.S. Government. The U.S. Government is authorized to reproduce and distribute reprints for Government purposes notwithstanding any copyright notation herein.

## Appendix

**Table:** Codebook for Manual Coding.

Action	Target
Support/Disparage/Comment on	Candidate 1
	Candidate 2
	Candidate 3
	Candidate 4
	Candidate 5
Agree with	Role 1: Spy A
	Role 2: Truth-teller A
	Role 3: Truth-teller B
	Role 4: Spy B
	Role 5: Truth-teller C
Justify (i.e., provide evidence/reasoning to support their own statement)	The group
Question (i.e., ask a question but not for another player’s opinion)	
Ask for opinion (i.e., ask for another player’s opinion)	
Change mind (i.e., change mind after the note presentation)	
Confusion (i.e., express confusion about whom to hire)	
